# The Intracellular Transport and Secretion of Calumenin-1/2 in Living Cells

**DOI:** 10.1371/journal.pone.0035344

**Published:** 2012-04-13

**Authors:** Qiao Wang, Hui Feng, Pengli Zheng, Birong Shen, Liang Chen, Lin Liu, Xiao Liu, Qingsong Hao, Shunchang Wang, Jianguo Chen, Junlin Teng

**Affiliations:** 1 State Key Laboratory of Bio-membrane and Membrane Bio-engineering and Key Laboratory of Cell Proliferation and Differentiation of the Ministry of Education, College of Life Sciences, Peking University, Beijing, China; 2 Center for Theoretical Biology, Peking University, Beijing, China; CNRS, France

## Abstract

Calumenin isoforms 1 and 2 (calu-1/2), encoded by the *CALU* gene, belong to the CREC protein family. Calu-1/2 proteins are secreted into the extracellular space, but the secretory process and regulatory mechanism are largely unknown. Here, using a time-lapse imaging system, we visualized the intracellular transport and secretory process of calu-1/2-EGFP after their translocation into the ER lumen. Interestingly, we observed that an abundance of calu-1/2-EGFP accumulated in cellular processes before being released into the extracellular space, while only part of calu-1/2-EGFP proteins were secreted directly after attaching to the cell periphery. Moreover, we found the secretion of calu-1/2-EGFP required microtubule integrity, and that calu-1/2-EGFP-containing vesicles were transported by the motor proteins Kif5b and cytoplasmic dynein. Finally, we determined the export signal of calu-1/2-EGFP (amino acid positions 20–46) and provided evidence that the asparagine at site 131 was indispensable for calu-1/2-EGFP stabilization. Taken together, we provide a detailed picture of the intracellular transport of calu-1/2-EGFP, which facilitates our understanding of the secretory mechanism of calu-1/2.

## Introduction

Human calumenin (calu), a CREC protein family member, is encoded by the *CALU* gene (NCBI GeneID: 813) [1], [2], which is mapped on chromosome 7q32 [3]. Two alternative spliced variants of the *CALU* gene are identified as calu-1 and calu-2 (also known as crocalbin) [4]. The two isoforms have equal lengths (315 amino acids), with exons 3 and 4 exchanged [5], and are ubiquitously expressed in human tissues [6]. Both calu-1 and -2 contain an N-terminal signal sequence (19 amino acids) and seven EF-hand domains for binding Ca^2+^ [7]. Previous reports show that they localize to the secretory pathway and are secreted into the extracellular space [8]–[10], while some researches insist that calu-1/2 contained an ER-retaining signal HDEF at the C-terminus, and are retained in the ER lumen [11]–[13]. Besides, proline at the +2 position from the predicted signal peptide cleavage site of calu-1/2 [6] acts as an export signal to mediate calu-1/2 secretion [14].

Extracellularly, calu-1 is reported to interact with the serum amyloid P component in the presence of Ca^2+^, indicating its possible role in amyloidosis [15]. Calu-1 may also be involved in autocrine and paracrine signaling since it decreases the expression level of septin 2 and actin fragments as well as regulating the cell cycle in fibroblasts [16]. Recently, thrombospondin-1, a secreted glycoprotein, is reported to form a complex with calu-1, suggesting that calu-1/2 play a potential role in hemostasis and thrombosis [2].

Despite the initial characterization of calu-1/2, many aspects are still elusive, including their intracellular transport and secretory process. Here, we used calu-1/2-EGFP, in which the EGFP would possibly mask the C-terminal HDEF retention signal, to study its secretion process. We reported the translocation of calu-1/2-EGFP into the lumen of the endoplasmic reticulum (ER), visualized their intracellular transport in the vesicles, and showed the secretion of calu-1/2-EGFP through either “secretion after attachment” or “secretion after accumulation”. To investigate the underlying transport mechanism, we determined the roles of cytoskeleton network and motor proteins on the intracellular transport and secretion of calu-1/2-EGFP. Furthermore, we identified that Kif5b and cytoplasmic dynein were the motors that were responsible for their microtubule-dependent trafficking. Finally, mutational analysis revealed the export signal of calu-1/2-EGFP and amino acid point crucial for the calu-1/2-EGFP stability.

## Results

### Calu-1/2 are translocated into the ER lumen and secreted

**Figure 1:**
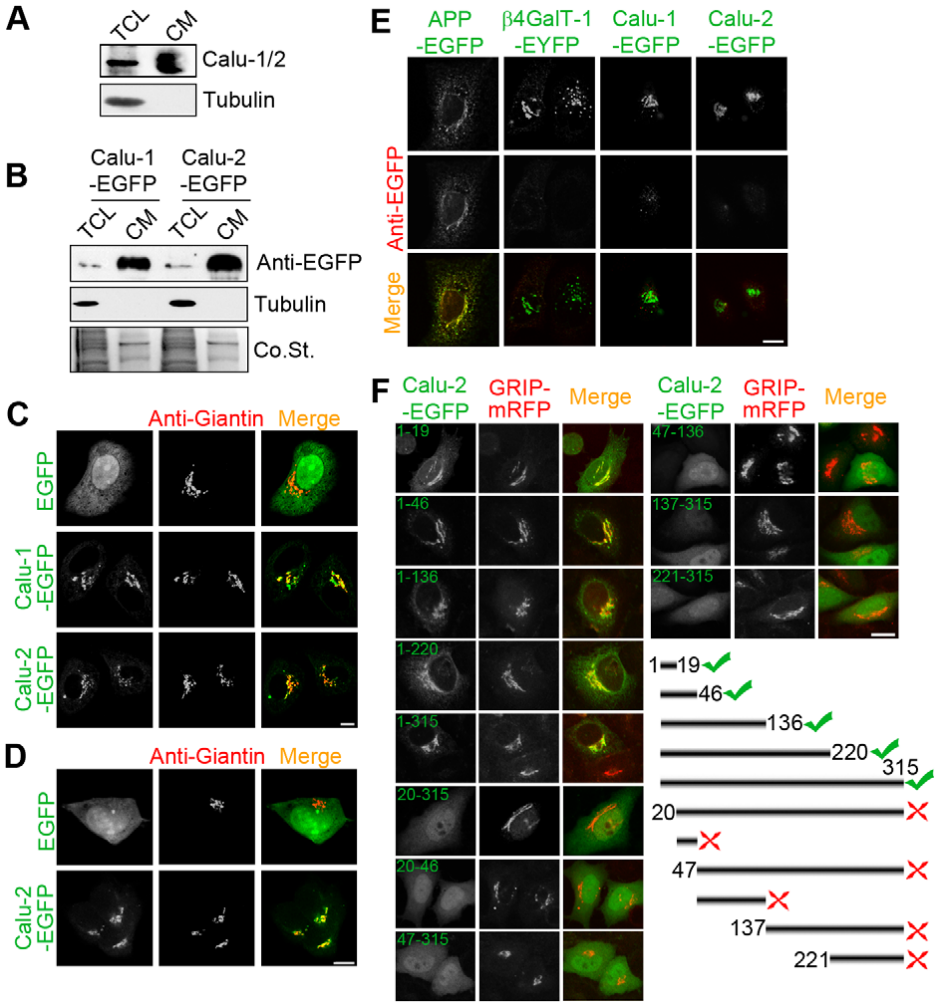
Calu-1/2 are translocated into the lumen and secreted. (A) Western blotting analysis of endogenous calu-1/2 from total cell lysate (TCL) and cultured medium (CM) of HeLa cells. (B) Western blotting analysis of overexpressed calu-1/2-EGFP of HeLa cells. Samples of TCL and CM were separated by SDS-PAGE and probed with anti-EGFP and anti-tubulin antibodies. Coomassie Blue staining (Co.St.) was used as a loading control of CM. (C) Immunofluorescence of EGFP or Calu-1/2-EGFP overexpressing HeLa cells probed with anti-Giantin antibody. Bar, 10 µm. (D) Immunofluorescence of EGFP or Calu-2-EGFP overexpressing HEK293T cells probed with anti-Giantin antibody. Bar, 10 µm. (E) HeLa cells were immunofluorescently labeled by the indicated antibodies after being permeabilized by digitonin. Scale bar, 10 µm. (F) Subcellular localization of 11 EGFP-fusion proteins of calu-2 transcripts in HeLa cells, which were co-transfected with GRIP-mRFP and permeabilized by Triton X-100 after fixation. Numbers indicate the amino acid positions in calu-2. In the cartoon, transcripts which localized on the secretory pathway are marked by green ticks. Red crosses indicate the transcripts that are smeared in HeLa cells. Scale bar, 10 µm.

With our produced antibody, which recognize both calu-1 and calu-2 isoforms (data not shown), we found that a great deal of calu-1/2 were detected in the cultured medium (Figure 1A). However, immunofluorescence assay showed that calu-1/2 localized throughout the cell in an ER-like manner rather than accumulated at the Golgi apparatus both in HeLa and HEK293T cells (Figure S1). In order to specifically study the secretory process of calu-1/2, we used calu-1/2-EGFP, in which the C-terminal ER-retaining signal HDEF was disrupted by the EGFP tag. A majority of calu-1/2-EGFP fusion proteins were detected in the cultured medium as expected (Figure 1B). Meanwhile, calu-1/2-EGFP accumulated at the Golgi apparatus both in HeLa and HEK293T cells (Figure 1C and 1D), suggesting that the ER-retaining pathway was blocked.

We then used digitonin, which selectively permeabilizes the plasma membrane but not the intracellular membranes [17], to treat the cells after fixation. Under this treatment, GM130, which peripherally attaches to the cytoplasmic surface of the Golgi apparatus, was detected by its antibody, whereas the ER luminal protein PDI could not be recognized by its own antibody (Figure S2). Similarly, the cytosolic EGFP tag of APP-EGFP fusion protein was immunolabeled by anti-EGFP antibody, whereas β4GalT-1-EGFP, whose EGFP is on the luminal side of the ER and the Golgi apparatus, was not immunolabeled (Figure 1E). Also, anti-EGFP antibody did not label the overexpressed calu-1/2-EGFP by immunofluorescence after digitonin treatment (Figure 1E), suggesting that calu-1/2 localized in the lumen of the ER and the Golgi apparatus. Furthermore, to determine the signal responsible for the entry of calu-1/2 into the lumen, we generated several constructs of calu-2 and visualized their subcellular localization, and found that the 19 amino acids on the N-terminal were necessary and sufficient for the Golgi localization of calu-2-EGFP (Figure 1F). These results, together with the previous report that the N-terminal 19 amino acids of calu-1 are cleaved [14], suggest that calu-1/2-EGFP are secreted to the extracellular space, and that the N-terminal 19 amino acids act as a signal peptide, which docks calu-1/2-EGFP to the ER and then translocates them into the lumen of the endomembrane system.

### Intracellular transport of calu-1/2-EGFP

**Figure 2:**
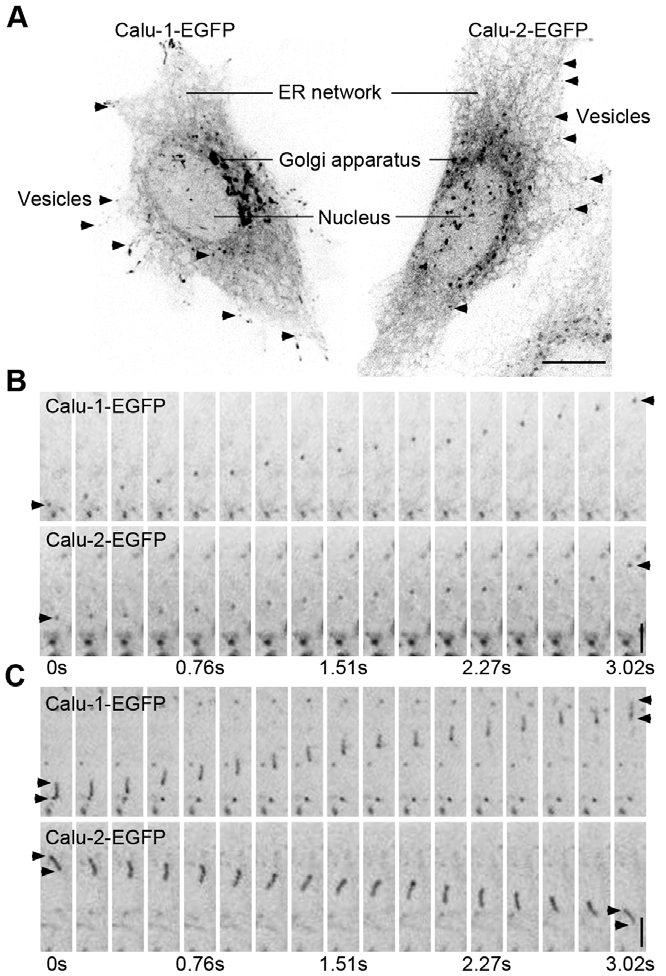
Time-lapse imaging of the intracellular transport of calu-1/2-EGFP. (A) Representative images of HeLa cells overexpressing calu-1/2-EGFP. Calu-1/2-EGFP fusion proteins localize in the Golgi apparatus and the ER networks, with the nuclei indicated. Vesicles are indicated by arrowheads. Scale bar, 10 µm. (B–C) Representative images of the intracellular transport of calu-1/2-EGFP. (B) Movements of single vesicles (indicated by one arrowhead) were tracked, and movements of rod-shaped vesicles (indicated by two arrowheads) are shown in (C). Scale bar, 1 µm.

To monitor the intracellular transport and secretory process of calu-1/2-EGFP, we monitored the distribution and dynamic behavior of calu-1/2-EGFP in live HeLa cells (≥30 cells) using an Andor spinning disk confocal microscope system. Both calu-1-EGFP and calu-2-EGFP localized to the secretory pathway, including the ER network and the Golgi apparatus (Figure 2A). Calu-1/2-EGFP-containing vesicles were observed throughout the cytoplasm (Figure 2A). Some appeared to be spherical (Figure 2B), while others were rod-shaped and tubular (Figure 2C). These calu-1/2-EGFP-containing vesicles were intracellularly transported (Figure 2A–C; Movies S1 and S2).

**Figure 3:**
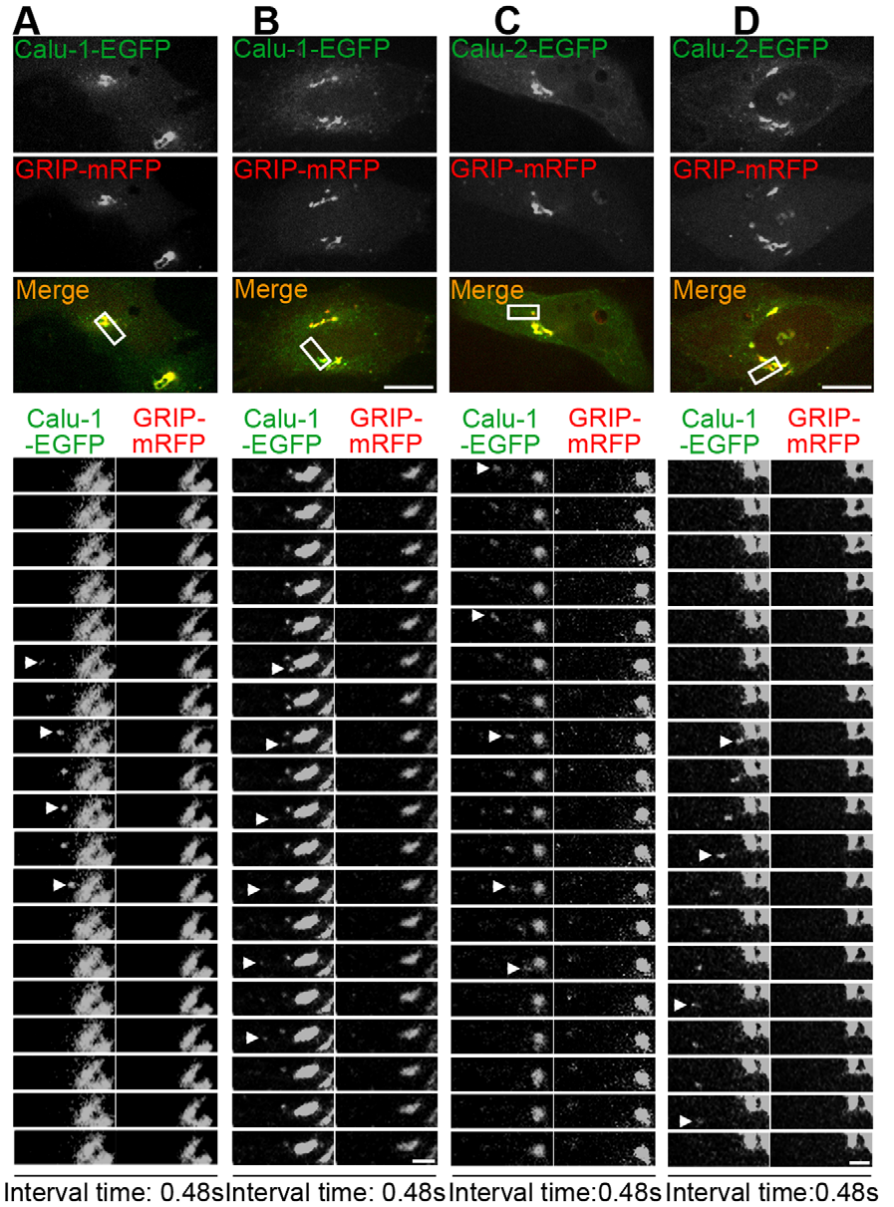
Calu-1/2-EGFP enter or exit the Golgi apparatus. (A–D) The representative HeLa cells transfected with calu-1-EGFP (A and B) and calu-2-EGFP (C and D) visualized by the Andor spinning disk confocal microscope system. The rectangles show magnified areas. Arrowheads point to those vesicles which enter (A and C) and exit (B and D) the Golgi apparatus. The time interval between two individual frames is 0.48 seconds. Scale bar, 1 µm.

Furthermore, to visualize the details of calu-1/2-EGFP intracellular transport, we co-expressed calu-1/2-EGFP and GRIP-mRFP (Golgi marker) in HeLa cells, and viewed the *in vivo* motion of calu-1/2-EGFP (≥5 cells). These calu-1/2-EGFP-containing vesicles entered or exited from the Golgi apparatus (Figure 3A–D). Taken together, calu-1/2-EGFP are distributed in the secretory pathway and extensively transported within the cell.

### Two types of calu-1/2-EGFP secretion

**Figure 4:**
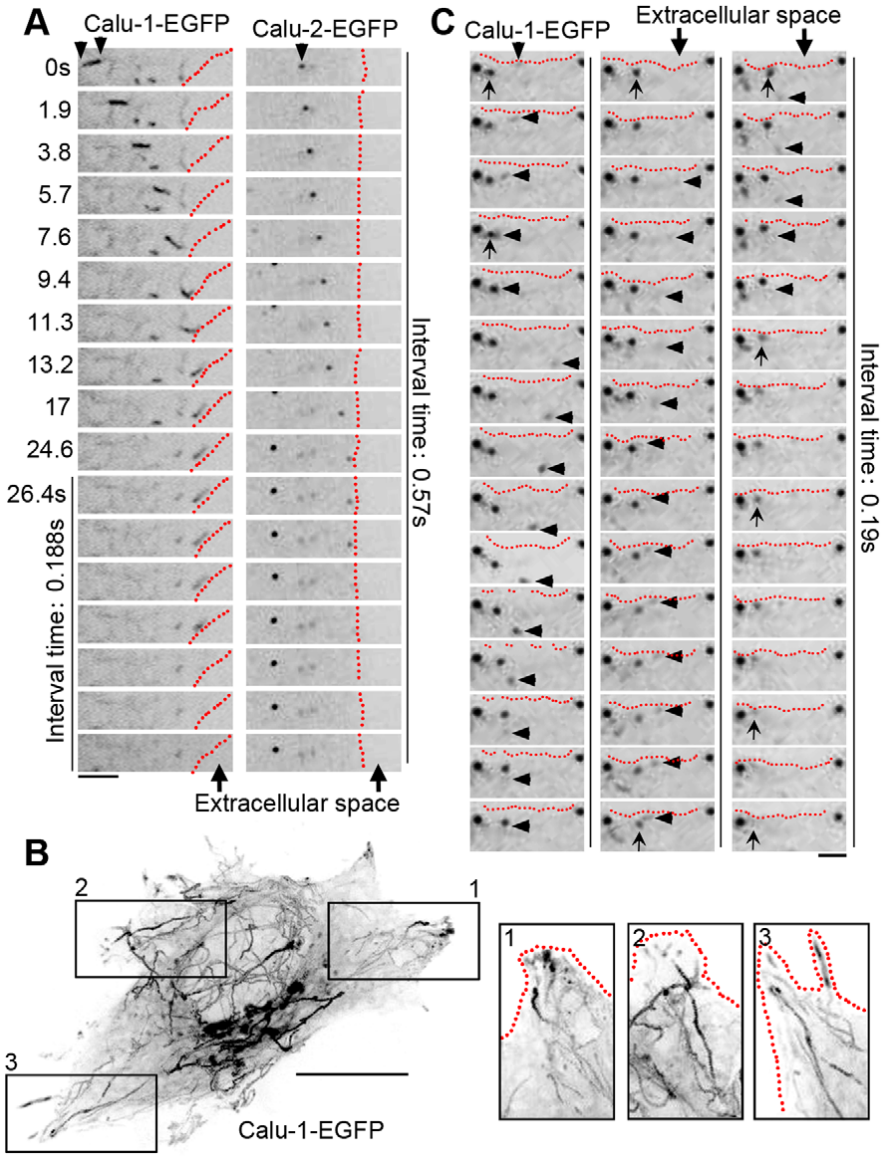
Calu-1/2-EGFP are secreted in two different types. (A) Time-lapse observation of calu-1-EGFP (left) and calu-2-EGFP (right) secretion in the “secretion after attachment” mode. Scale bar, 4 µm. (B) Vesicle tracks in calu-1-EGFP-expressing HeLa cells. Images were acquired at 0.188-second intervals. These sequential frames were overlaid as time projections to illustrate vesicle paths. The rectangles are magnified areas showing the accumulation of calu-1-EGFP in the cellular processes. Scale bar, 10 µm. (C) Time-lapse observation of calu-1-EGFP secretion in the “secretion after accumulation” mode. Note that the arrow head pointed calu-1-EGFP dots moved to the arrow pointed dot, and the arrow pointed dot vanished finally. Scale bar, 4 µm.

To further visualize the secretory process of calu-1/2-EGFP, we observed in more detail the *in vivo* behavior of calu-1/2-EGFP at the periphery of the cells, and found two main types of secretion. Some the calu-1/2-EGFP -containing vesicles moved toward the cell periphery (Figure 4A; Movies S1 and S2). One or two seconds later, the calu-1/2-EGFP signal faded rapidly and finally vanished, suggesting that the calu-1/2-EGFP proteins were secreted into the extracellular space. Interestingly, we noted another secretory manner, in which vesicles were transported towards the periphery of the cell and accumulated at cellular processes (Figure 4B; Movies S1 and S2). We also noted that these cell-tip-heading vesicles seemed to fuse with each other before being secreted together (Figure 4C), indicating that calu-1/2-EGFP proteins were secreted collectively after their accumulation in the tip of cells.

### Calu-1/2-EGFP-containing vesicles are transported by Kif5b and cytoplasmic dynein along microtubules

**Figure 5:**
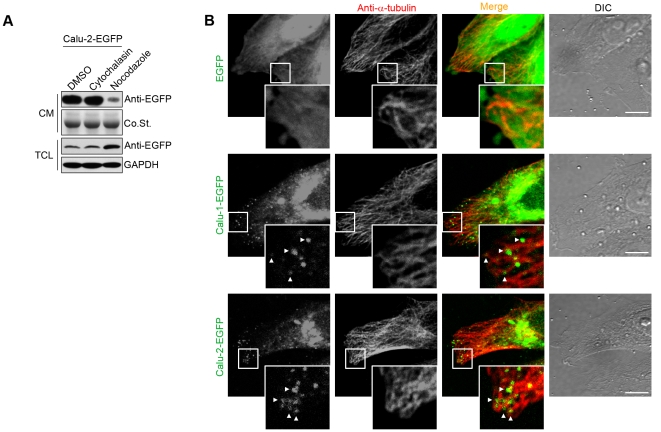
The transport of calu-1/2-EGFP-containing vesicles are microtubule-dependent but actin-independent. (A) Western blotting analysis in TCL and CM of the calu-2-EGFP transfected cells treated with DMSO, nocodazole or cytochalasin. (B) Immunofluorescence of calu-2-EGFP expressing HeLa cells using anti-α-tubulin antibody. Arrow heads indicate calu-2-EGFP dots spreading along the microtubules to cellular processes. The rectangle areas were amplified. Scale bar, 10 µm.

Having visualized the intracellular transport and secretion of calu-1/2-EGFP, we then further investigated the molecular mechanism of calu-1/2 transport. To determine whether the secretion of calu-1/2-EGFP depends on the cytoskeleton, we treated the calu-1/2-EGFP-overexpressing cells with different drugs to interfere with the polymerization of microtubules or actin filaments. Nocodazole binds to β-tubulin and blocks tubulin self-assembly, thus interfering with the polymerization of microtubules [18]. Cytochalasin blocks both assembly and disassembly of individual actin monomers by binding to the plus ends of microfilaments, thus inhibiting the normal polymerization and elongation of actin filaments [19]. After nocodazole treatment, the protein level of calu-2-EGFP markedly deceased in the cultured medium, while increased a little in the cell lysate (Figure 5A). On the contrary, cytochalasin treatment did not alter the secretory level (Figure 5A). Besides, using immunofluorescence assay, we found calu-1/2-EGFP green dots localized along microtubules to cellular processes (Figure 5B and S3). Therefore, the secretion of calu-1/2-EGFP is microtubule dependent but is not affected by the integrity of actin filament.

**Figure 6:**
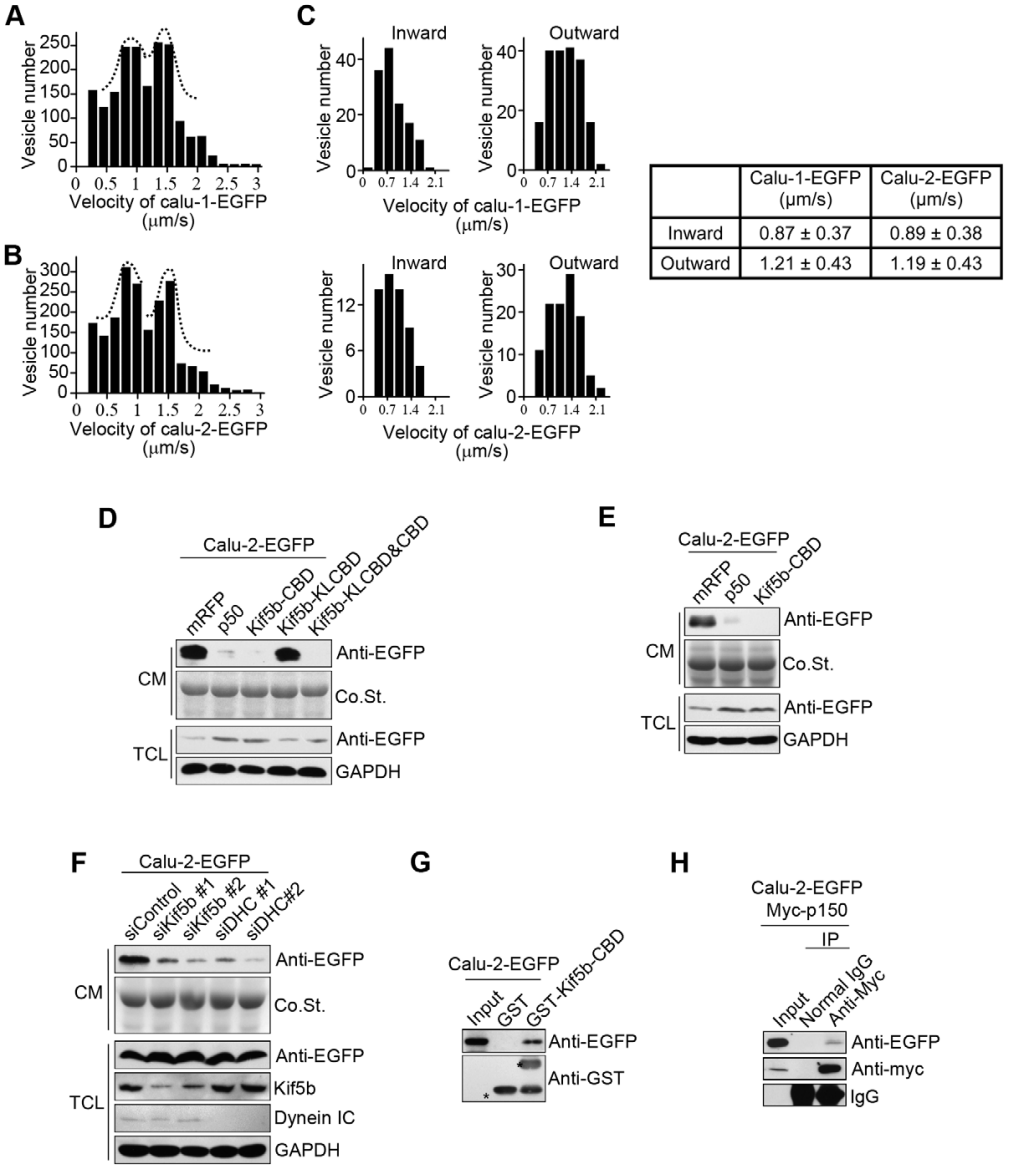
Calu-1/2-EGFP-containing vesicles are transported by Kif5b and cytoplasmic dynein. (A–B) Histograms show the distribution of velocities of calu-1/2-EGFP-containing vesicles in HeLa cells. The dashed lines indicate that most vesicles move at 0.7–1 µm/s and 1.1–1.5 µm/s. Approximately 2000 dots (≥10 cells) were measured for each group. (C) The inward and outward velocity of calu-1/2-EGFP-containing vesicles. (D) Western blotting analysis of calu-2-EGFP secretory level in HeLa cells co-transfected with the indicated dominant negative vectors. (E) Western blotting analysis of calu-2-EGFP secretory level in HEK293T cells co-transfected with the indicated dominant negative vectors. (F) Western blotting analysis of calu-2-EGFP secretory level of Kif5b or cytoplasmic dynein knockdown HeLa cells. (G) GST pull-down analysis of calu-2-EGFP transfected cells using GST or GST-Kif5b-CBD. (H) Immunoprecipitation assay of calu-2-EGFP and myc-p150 overexpressing HEK293T cells using normal IgG or anti-myc antibody.

To determine which motor protein mediates the calu-1/2-EGFP transport, we measured the velocities of calu-1/2-EGFP containing vesicles. We calculated the velocities of more than 2000 dots (≥10 cells), and found that calu-1-EGFP-containing vesicles moved at an average velocity of 1.15±0.55 µm/s (Figure 6A), similar to the average velocity of the vesicles containing calu-2-EGFP (1.12±0.56 µm/s) (Figure 6B). Although some vesicles moved rapidly, reaching 3 µm/s, frequency count analysis showed that most vesicles of both calu-1-EGFP and calu-2-EGFP moved at 0.7–1 µm/s or 1.1–1.5 µm/s (Figure 6A and 6B). These two peak values indicate that calu-1/2-EGFP-containing vesicles are transported by two motors with different motilities.

In eukaryotes, there exist two kinds of microtubule motor proteins, kinesin and dynein, which widely participate in vesicle transport [20], [21]. The velocity of kinesin heavy chain (Kif5b) motor and cytoplasmic dynein both varied a lot in previous reports, but many researches showed that both Kif5b and cytoplasmic dynein move at ∼1 µm/s *in vivo* [22]–[27]. Considering that Kif5b and cytoplasmic dynein moved at different directions, we measured the inward and outward velocity of calu-1/2-EGFP-containing vesicles separately. We found that calu-1/2-EGFP-containing vesicles heading the Golgi apparatus moved at ∼0.9 µm/s, while those moving towards the cell periphery moved at ∼1.2 µm/s (Figure 6C). These two velocities fit well with the two peaks and are similar to the previously reported velocities of Kif5b and cytoplasmic dynein, suggesting that calu-1/2-EGFP-containing vesicles are transported by Kif5b and cytoplasmic dynein at different speed.

To further confirm our hypothesis, we performed dominant negative experiments. Overexpressed p50, one subunit of the dynactin complex, acts as a dominant-negative inhibitor of dynein-dynactin, and disrupts cytoplasmic dynein-dependent organelle movement [28]. Kif5b interacts with its cargoes through kinesin light chain via the kinesin-light-chain binding domain (KLCBD, amino acids 760–830), or through cargo binding domain (CBD, amino acids 831–964). Overexpression of the KLCBD and CBD of Kif5b cause a dominant negative phenotype of Kif5b, through their competitive cargo binding effect [29], [30]. In the cells transfected with p50, Kif5b-CBD or Kif5b-KLCBD&CBD, the protein level of calu-2-EGFP in the cultured medium reduced significantly (Figure 6D and 6E). Besides, overexpression of Kif5b-KLCBD did not change the secretion level (Figure 6D), suggesting that the CBD, but not the KLCBD of Kif5b, is involved in the recognition of calu-1/2-EGFP-containing vesicles. However, we could not exclude the possibility that the overexpressed Kif5b-CBD directly inhibited the activity of endogenous Kif5b through the binding to its motor domain [31]. Therefore, we knocked down Kif5b, detected the reduced secretion level of calu-2-EGFP (Figure 6F), further suggesting that Kif5b participated in transporting calu-1/2-EGFP. Similarly, the knockdown of cytoplasmic dynein also reduced the secretion level of clau-2-EGFP (Figure 6F). Collectively, these data suggested that cytoplasmic dynein and Kif5b were involved in the transport of calu-1/2-EGFP-containing vesicles.

**Figure 7:**
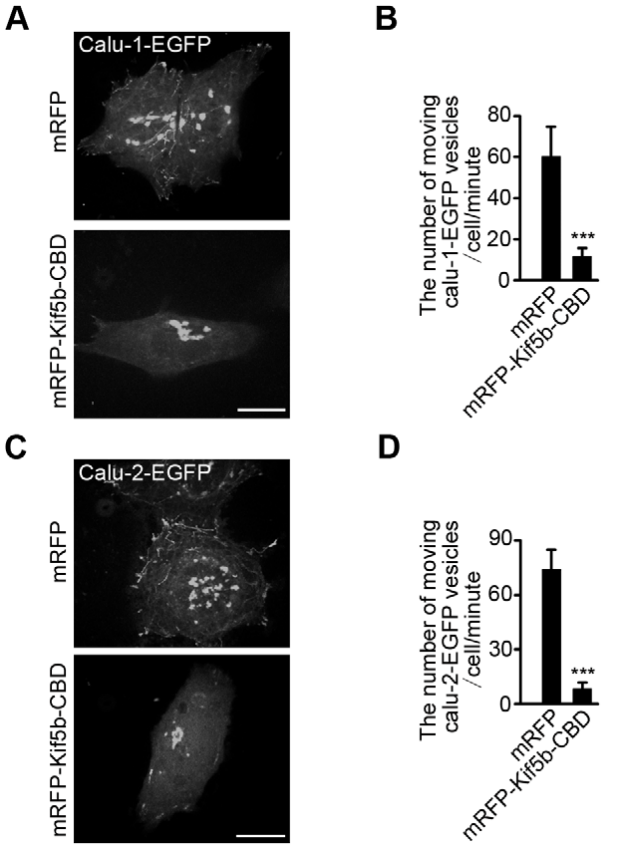
Dominant negative effects of Kif5b on the calu-1/2-EGFP-containing vesicle trafficking in HeLa cells. (A) The representative picture shows the tracks of moving vesicles containing calu-1-EGFP in the presence of mRFP or mRFP-Kif5b-CBD. These tracks of moving vesicles are real-time projections of individual frames over 3 minutes. (B) The numbers of moving calu-1-EGFP-containing vesicles per minute corresponding to (A) were manually counted (≥10 cells in each group). (C) The representative picture shows the tracks of moving vesicles containing calu-2-EGFP in the presence of mRFP or mRFP-Kif5b-CBD. These tracks of moving vesicles are real-time projections of individual frames over 3 minutes. (D) The numbers of moving calu-2-EGFP-containing vesicles per minute corresponding to (C) were manually counted (≥10 cells in each group). Scale bar, 10 µm.

To further demonstrate the motor-cargo association, we performed pull-down and immunoprecipitation analysis. Calu-2-EGFP was pulled down by GST-Kif5b-CBD (Figure 6G). Immunoprecipitation assay also confirmed the association between cytoplasmic dynein complex and calu-2-EGFP (Figure 6H). Meanwhile, overexpressed Kif5b-CBD dramatically decreased the number of moving calu-1/2-EGFP vesicles (Figure 7; Movies S3, S4, S5, S6). Taken together, calu-1/2-EGFP-containing vesicles are transported by Kif5b and cytoplasmic dynein, and the Kif5b-CBD and dynactin are necessary for calu-1/2-EGFP secretion.

### Export signal of calu-1/2-EGFP

**Figure 8:**
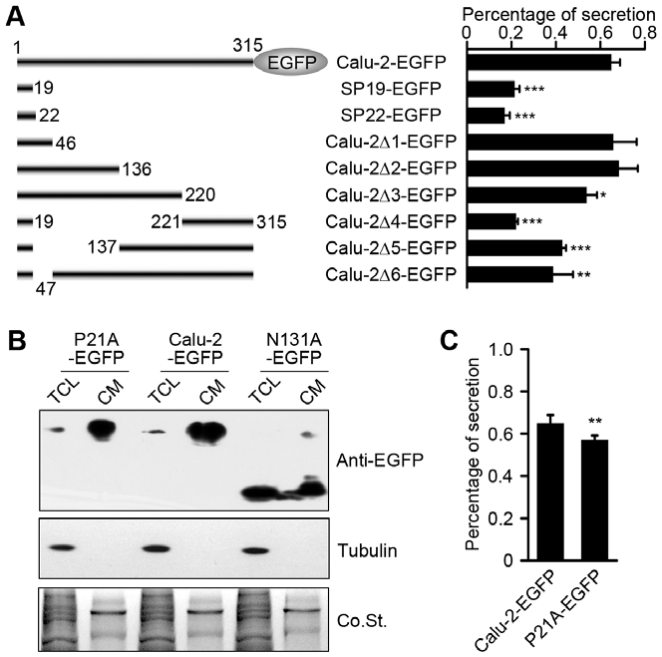
The export signal for calu-1/2-EGFP. (A) Percentage secretion of different calu-2 truncated constructs. The right column shows the percentage secretion of nine constructs showed in the left column. These secretory rates were calculated according to the Western blotting results (data not shown, three independent experiments). (B) Point mutation effects on calu-2-EGFP secretion. Western blotting analysis showed calu-2-EGFP secretory level of two point mutations. Samples were probed with anti-EGFP and anti-tubulin antibodies. Co.St. was used as a loading control of CM. (C) The percentage secretion of calu-2-EGFP mutation constructs shown in (B) (three independent experiments).

Soluble cargo proteins in the intracellular lumen are exported by either bulk flow or receptor-mediated transport [32]–[37]. To investigate whether calu-1/2-EGFP is passively or actively exported into the extracellular space, we expressed the fusion proteins SP19-EGFP, which are the EGFP-tagged N-terminal 19 amino acids of calu-1/2 (Figure 8A). If the secretion of calu-1/2-EGFP is through bulk flow, SP19-EGFP should have the same percentage secretion as full-length calu-1/2-EGFP. However, we found that the percentage secretion of SP19-EGFP was dramatically decreased compared to the calu-2-EGFP (Figure 8A). To exclude the possibility that N-terminal signal peptide cleavage of SP19-EGFP was disrupted, thus leading to its retention, we further studied SP22-EGFP. SP22-EGFP showed similar percentage secretion to SP19-EGFP (Figure 8A), suggesting that calu-1/2 may not be secreted through bulk flow but *via* receptor-mediated secretory pathway.

Next, to identify the export signal for the calu-1/2 secretion, we constructed transcripts of calu-2(Δ1–Δ6)-EGFP (Figure 8A). The percentage secretions of calu-2(Δ1–Δ3)-EGFP were nearly the same as that of calu-2-EGFP; on the contrary, calu-2(Δ4–Δ6)-EGFP exhibited a significant reduction of secretory level (Figure 8A). These data suggested that a sorting signal, which was crucial for the receptor-mediated export of calu-1/2-EGFP, was located between the amino acid positions 20 and 46. Furthermore, within the sorting signal, we noted that the proline at site 21 was reported to be an ER export signal [14]. To determine whether this site alone plays a vital role in calu-1/2-EGFP secretion, we mutated it to alanine, and measured the percentage secretion. The percentage secretion of calu-2-P21A-EGFP was only slightly decreased compared with the control (Figure 8B and 8C), suggesting that the proline at site 21 is not the only key element for calu-1/2-EGFP secretion but may contribute to the integrity of the sorting signal between amino acids 20–46.

### Asparagine at site 131 is important for calu-1/2-EGFP stability

The asparagine at site 131 is predicted to be an N-glycosylation site [8], [11]. To test this hypothesis, we investigated whether this site affects calu-1/2-EGFP secretion. Surprisingly, we found that when the asparagine at position 131 was mutated to alanine, calu-2(N131A)-EGFP was degraded extensively both in the intracellular and extracellular space (Figure 8B), suggesting that the N-glycosylation at this site plays an important role in calu-1/2-EGFP stability, which needs further investigation.

## Discussion

**Figure 9:**
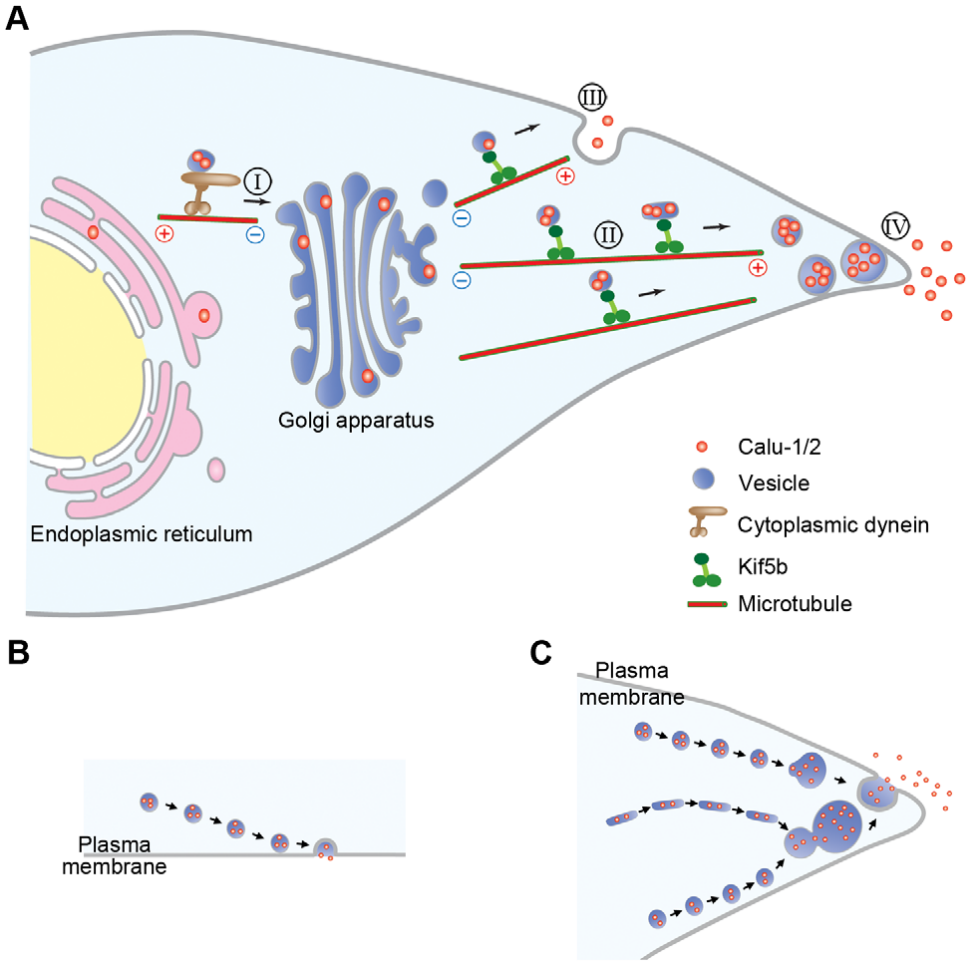
Schematic diagram illustrating the intracellular transport and secretory process of calu-1/2-EGFP. (A) In the eukaryotic cell, calu-1/2-EGFP are simultaneously synthesized and translocated into the lumen of the endoplasmic reticulum, and then exit the ER with the help of the motor protein cytoplasmic dynein, being enclosed in vesicles (step I). After entering the Golgi apparatus, calu-1/2-EGFP are glycosylated and then transported within the secretory vesicles along microtubules by Kif5b to the periphery of the cell (step II). Then, calu-1/2-EGFP are secreted to the extracellular space either directly after attaching to the cell membrane (step III) or first accumulate in cellular processes and then are released together (step IV). (B) Model for “secretion after attachment”. (C) Model for “secretion after accumulation”.

In this study, we showed that calu-1/2-EGFP are translocated into the lumen with the help of their signal peptide, transported intracellularly in vesicles, and finally secreted into the extracellular space (Figure 9), during which the sorting signal on calu-1/2 is indispensable. We examined the velocities of calu-1/2-EGFP-containing vesicles, and identified the motor proteins that participate in powering calu-1/2-EGFP-containing vesicles. Most interestingly, besides the traditional mode of secretion, we also observed another type of secretion, in which calu-1/2-EGFP-containing vesicles accumulated in cellular processes and were secreted collectively.

During the secretory process, protein with an N-terminal signal peptide is synthesized in the rough ER-bound ribosomes and then translocated into the ER lumen. After proper folding and modification, the protein is delivered to the Golgi apparatus for further modification and finally released into the extracellular space through vesicular transport [38]. Among the various motor proteins responsible for the intracellular transport of diverse cargoes, the microtubule motor protein cytoplasmic dynein moves towards the minus end of the microtubules, participating in anterograde transport from the ER to the Golgi apparatus [21], [39], and Kif5b, a microtubule plus end-directed motor, is responsible for post-Golgi traffic [20], [40]. Previous reports about the velocities of Kif5b and cytoplasmic dynein *in vivo* varied a lot, from 0.2 µm/s to larger than 3 µm/s [22]–[27], [41]–[44]. In our experiments, calu-1/2-EGFP-containing vesicles also moved at different speeds, with some moved at 0.3–0.5 µm/s while some fast vesicles reached 3 µm/s. This is consistent with previous reports. Besides, we found that calu-1/2-EGFP-containing vesicles moved at ∼0.9 µm/s inward and ∼1.2 µm/s outward. We hypothesized that cytoplasmic dynein and Kif5b were responsible for the intracellular transport of calu-1/2-EGFP at different speeds. Using dominant negative and RNAi methods, we found that the blockage of Kif5b or cytoplasmic dynein significantly inhibited the secretion of calu-1/2-EGFP (Figure 6D–F). Moreover, GST pull-down and immunoprecipitation analysis further confirmed the association between Kif5b/cytoplasmic dynein and calu-1/2-EGFP-containing vesicles (Figure 6G–H). Therefore, it is possible that cytoplasmic dynein mediates the ER-to-Golgi transport of calu-1/2-EGFP at a relatively slower speed of ∼0.9 µm/s, while Kif5b executes the transport of calu-1/2-EGFP-containing vesicles at ∼1.2 µm/s during post-Golgi transport (Figure 9). Also, we do not exclude the possibility that calu-1/2-EGFP-containing vesicles are bidirectionally transported through spatial-temporal regulation of Kif5b and dynein cooperation [45], [46], and some other motor proteins may also be involved in the transport of calu-2-EGFP.

In our experiments, we found two types of secretion of calu-1/2-EGFP. In the model of “secretion after attachment” (Figure 9B), which is traditionally reported [42], the release of the secretory product is thought to be regulated by the network of actin filaments. It was suggested that the secretory vesicles traveling to the periphery of the cell are inhibited to fuse with each other and the plasma membrane by a dense web of microfilaments, and the stimulation of the rapid depolymerization of these actin filaments leads to the following vesicle fusion and secretion [47], [48]. However, our results show that the disruption of actin filament network does not enhance the secretory level of calu-2-EGFP (Figure 5A), suggesting most calu-1/2-EGFP may not use this secretory mode, but are secreted through the model of “secretion after accumulation” (Figure 9C), which is also observed in mRNA trafficking [49]. Moreover, the projection of the green fluorescent dots (Figure 4B) not only stands for the trafficking paths of calu-1/2-EGFP-containing vesicles, but may also represent the microtubule tracks. This is further supported by the immunostaining data, in which microtubules pointed to cellular processes with calu-2-EGFP dots aligning along them (Figure 5B and S3). Therefore, we propose that it is the microtubules directed towards cellular processes that facilitate the accumulation of calu-1/2-EGFP-containing vesicles. Previous reports show that exocytotic vesicles are targeted to the ELKS-containing patches where MT plus ends are attached [42], [50]. So it is possible that the cellular processes are abundant with ELKS and attract calu-1/2-EGFP-containing vesicles.

Soluble proteins with ER exit signals are recognized by transmembrane receptors, which recruit the coat proteins, and mediate the transport from the ER to the Golgi apparatus, and finally to the cell periphery [32], [33], [35]. Besides, some proteins without ER exit signals also enter secretory vesicles and are secreted [34], [37]. Our data indicate that calu-1/2-EGFP are secreted through the receptor-mediated pathway, with their exit signal located at amino acid positions 20–46.

In addition, we identified the asparagine at position 131, which was predicted to be a glycosylation site [8], [11], as essential for calu-1/2-EGFP stability. The asparagine to alanine mutation lead to extensive degradation of calu-2-EGFP both intracellularly and extracellularly. This can be explained by two possibilities: either the glycosylation at this site stabilize calu-2-EGFP, protecting it from being cleaved by protease, or the mutation itself leads to misfolding of calu-2(N131A)-EGFP, its stuck in the ER and subsequent degradation via the ERAD pathway. Further experiments are needed to uncover the detailed mechanism.

## Materials and Methods

### Cell culture and transfection

HeLa [30] and HEK293T cells [51] were cultured in Dulbecco's modified Eagle's medium (DMEM) (GIBCO BRL) supplemented with 10% FBS at 37°C with 5% CO_2_ as described previously [30], [52]. Calcium phosphate precipitation or jetPEI (Polyplus) was used to transiently transfect HeLa and HEK293T cells according to previous reports [53].

### Antibodies

Mouse anti-human calu-2 polyclonal antibody (pAb) and rabbit anti-EGFP pAb were developed using prokaryotic expressed and purified protein GST-calu-2. Mouse anti-human calu-2 pAb recognizes both the calu-1 and -2 isoforms (data not shown). Anti-Kif5b antibody was produced previously [54]. Anti-PDI (ab2792), anti-GAPDH (ab8245), anti-GM130 (ab1299) and anti-Giantin (ab24586) were from Abcam. Anti-α-tubulin (DM1α) was from Sigma. Anti-myc antibody (M047-3) was from MBL. The secondary antibodies Alexa Fluor 488-/568-conjugated goat anti-mouse/rabbit IgG antibodies (Invitrogen) and HRP-conjugated antibodies (Invitrogen) were used in immunofluorescence and Western blotting analysis following the manufacturers' instructions.

### Vector construction

Calu-1/2 transcripts were amplified from HeLa cDNA and inserted into the *Eco*RI/*Sal*I site on the pEGFP-N3 vector (Clontech). Two point mutations of calu-2, P21A and N131A, were also constructed into the pEGFP-N3 vector (see supplementary material Table S1 for primer information). APP-EGFP vector was obtained by cloning APP into the *Xho*I/*Sal*I site on the pEGFP-N3 vector. The β4GalT-1-EYFP encodes an EYFP-tagged fusion protein containing the N-terminal 81 amino acids of human β4GalT-1. GRIP-mRFP encodes a fusion protein to mark the Golgi apparatus as previously reported [55]. Vector p50-EGFP was from Prof. CM Zhang (Peking University), and p50 was then amplified and inserted into the *Eco*RI/*Sal*I site on pmRFP-C1 (Clontech). Kif5b-CBD (amino acids 831–964), Kif5b-KLCBD (amino acids 760–830), and Kif5b-KLCBD&CBD were amplified from cDNA of HeLa cells, and inserted into the *Eco*RI/*Sal*I site on pmRFP-C1 (Clontech). Myc-p150 [56] was constructed into the BamHI/XhoI site of pcDNA3.1 vector. See Table S1 for primer information.

### Immunoblotting analysis and percentage secretion

Proteins were separated by SDS-PAGE and then transferred onto PVDF membranes (Millipore) in a semidry transfer cell (Bio-Rad). The membranes were blocked and subsequently probed with primary and secondary antibodies as previously reported [57].

For detecting the protein level in the cultured medium of HeLa cells, we substituted the medium with the serum-free DMEM 24 hours after transfection, and collected the total cell lysate (TCL) and the corresponding serum-free cultured medium (CM) from each cell culture dish 48 hours after transfection. As for experiments in HEK293T cells, we changed the medium with serum-free medium 12 hours post transfection and collected for 24 hours. Both TCL and CM were compressed to 50 µL, and a fifth of the total sample (10 µL) was loaded and subjected to Western blotting. The intensity of each target band was quantified using Image J analysis software (NIH). The percentage secretion is the ratio of the band intensity in the CM to the total intensity of the target band in both TCL and CM. Besides, in some experiments, cells were treated with DMSO, nocodazole (2 µg/ml), and cytochalasin B (1 µg/ml) in serum-free medium, and the TCL and CM were collected 24 hours later for Western blotting analysis.

### Fluorescence microscopy and *time-lapse* imaging

Transfected HeLa cells were fixed with freshly prepared 4% paraformaldehyde in PBS, and then subjected to direct observation under a fluorescence microscope (Olympus) or a confocal microscope (Leica). For immunofluorescence, the fixed cells were incubated with antibodies after permeabilization (0.15% Triton X-100 in PBS) and blocking. For digitonin treatment, the cells were permeabilized with 25 µg/ml digitonin in KHM buffer on ice as previously reported [17], and then fixed with 4% paraformaldehyde. For live cell imaging, the transfected cells were subjected to spinning disk laser microscopy (Andor) 24 hours after transfection. Images were collected using Andor software.

### RNA interference

For knockdown experiment in HeLa cells, siRNAs targeting Kif5b and cytoplasmic dynein according to previously report [42] were synthesized, and transfected into the HeLa cells using Lipofectamine 2000 (Invitrogen) as the manufacture's instruction. Control scramble sequence were designed by GenePharma Co.,Ltd.

### Pull-down and immunoprecipitation

For pull-down analysis, the fusion protein GST-Kif5b-CBD was prokaryotically expressed and attached to glutathione-sepharose 4B beads (Amersham). The total cell lysate of calu-2-EGFP transfected HEK293T cell was then collected and incubated with the affinity column in lysis buffer (20 mM HEPES, 120 mM NaCl, 5 mM EGTA, 2 mM MgCl2 and 1 mM DTT, pH 7.4) at 4°C overnight. The combined protein was harvested and tested by Western blotting.

For immunoprecipitation assay, the calu-2-EGFP and myc-p150 transfected HEK293T cell was lysed in IP buffer (20 mM HEPES, 250 mM sucrose, 10 mM KCl, 1 mM EGTA, 1 mM EDTA, 1.5 mM MgCl2 and 1 mM DTT, pH 7.4) containing protease inhibitor cocktail (Roche). Then, nucleus were removed by centrifugation at 3,000 rpm for 5 minutes. The supernatant was further centrifugated at 8,000 rpm for 15 minutes to remove mitochondria. The remained cytosolic and membrane fraction was then incubated with anti-myc antibody at 4°C overnight. The protein samples that combined with the Protein A Sepharose (Amersham) were collected and subjected to Western blotting.

### Statistical analysis

Images analyzed using Andor software and Image J (NIH). Vesicle motility (with a velocity exceeding 0.3 µm/s), relative fluorescence intensity, and vesicle path projection were acquired as previously reported [24], [42]. The velocities of moving vesicles were measured using the Image J software (NIH). The distance that one dot moved every two frames was calculated, with dots tracked manually, and the corresponding velocity was measured according to the interval time (0.376 seconds). Approximately 2000 dots (≥10 cells) were measured for each group. As for the calculation of the number of moving vesicles, we manually counted the number of moving dots in each cells for one minute (≥10 cells in each group).

Results are presented as mean ± standard deviation. Comparisons were analyzed by unpaired, two-sided, independent student's t test with or without equal variance assumption, according to the result of an F test. * means p<0.05, ** means p<0.01, *** means p<0.001.

## Supporting Information

Figure S1 Immunofluorescence of HeLa and HEK293T cells probed with anti-calu-1/2 antibody or anti-Giantin antibody. Scale bar, 10 µm.
(TIF)

Figure S2 Immunofluorescence of HeLa cells probed with anti-GM130 antibody or anti-PDI antibody after digitonin or Triton X-100 treatment. Scale bar, 10 µm.
(TIF)

Figure S3 Immunofluorescence of EGFP or Calu-2-EGFP overexpressing HEK293T cells probed with anti-α-tubulin antibody. The rectangle areas were magnified. Scale bar, 10 µm.
(TIF)

Table S1 Sequence information of primers used.
(DOC)

Movie S1 **Intracellular transport of calu-1-EGFP-containing vesicles.** The video records the movement of calu-1-EGFP-containing vesicles in HeLa cells at 0.188-seceond interval for 38 seconds. The time and scale are indicated.
(AVI)

Movie S2 **Intracellular transport of calu-2-EGFP-containing vesicles.** The video records the movement of calu-2-EGFP-containing vesicles in HeLa cells for 38 seconds. The time and scale are indicated.
(AVI)

Movie S3 **Transport of calu-1-EGFP in HeLa cells overexpressing mRFP.** The video records the movement of calu-1-EGFP-containing vesicles in HeLa cells at 0.188-seceond interval for 38 seconds. The scale is indicated.
(AVI)

Movie S4 **Transport of calu-1-EGFP in HeLa cells overexpressing mRFP-Kif5b-CBD.** The video records the movement of calu-1-EGFP-containing vesicles in HeLa cells at 0.188-seceond interval for 38 seconds. The scale is indicated.
(AVI)

Movie S5 **Transport of calu-2-EGFP in HeLa cells overexpressing mRFP.** The video records the movement of calu-2-EGFP-containing vesicles in HeLa cells at 0.188-seceond interval for 38 seconds. The scale is indicated.
(AVI)

Movie S6 **Transport of calu-2-EGFP in HeLa cells overexpressing mRFP-Kif5b-CBD.** The video records the movement of calu-2-EGFP-containing vesicles in HeLa cells at 0.188-seceond interval for 38 seconds. The scale is indicated.
(AVI)
