# Supplementary figures and images for: The Intracellular Transport and Secretion of Calumenin-1/2 in Living Cells
Source: PLoS One. 2012 Apr 13;7(4):e35344. doi: 10.1371/journal.pone.0035344 (PMC3325945; doi:10.1371/journal.pone.0035344)

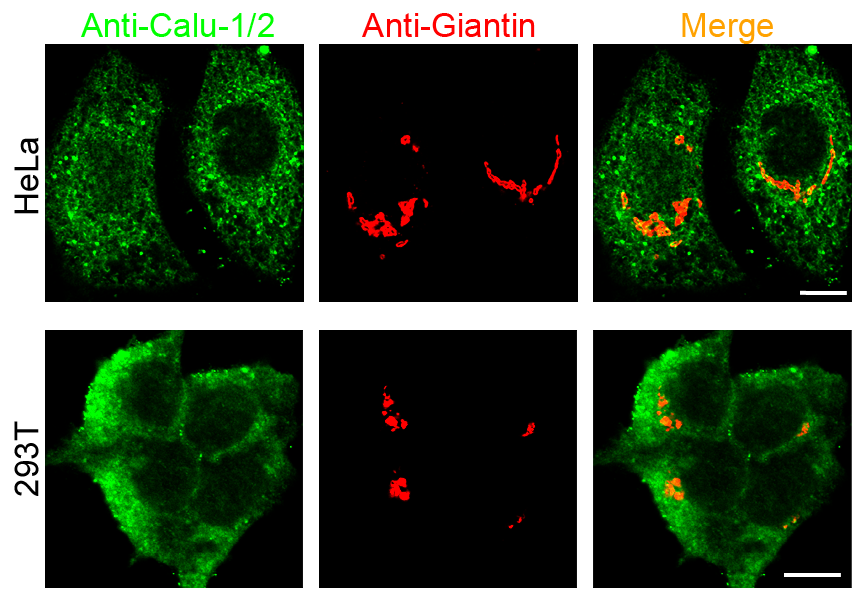

Supplement: Figure S1 — Immunofluorescence of HeLa and HEK293T cells probed with anti-calu-1/2 antibody or anti-Giantin antibody. Scale bar, 10 µm. (TIF) [file pone.0035344.s001.tif]

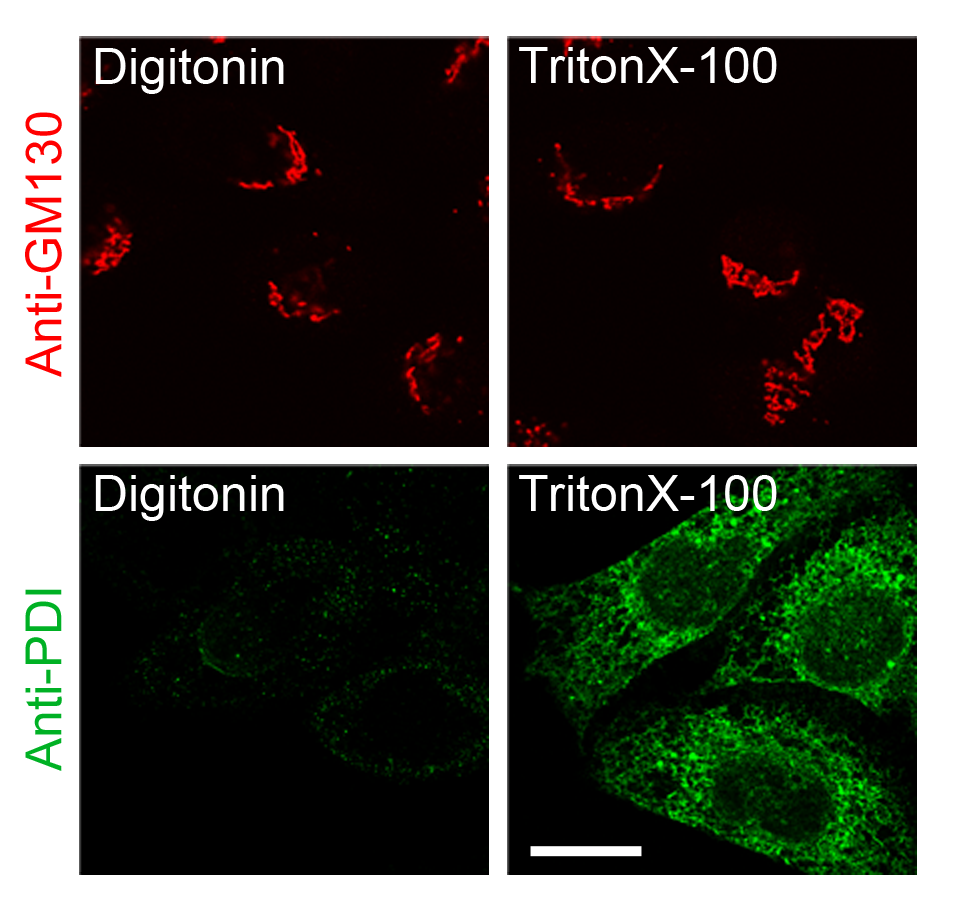

Supplement: Figure S2 — Immunofluorescence of HeLa cells probed with anti-GM130 antibody or anti-PDI antibody after digitonin or Triton X-100 treatment. Scale bar, 10 µm. (TIF) [file pone.0035344.s002.tif]

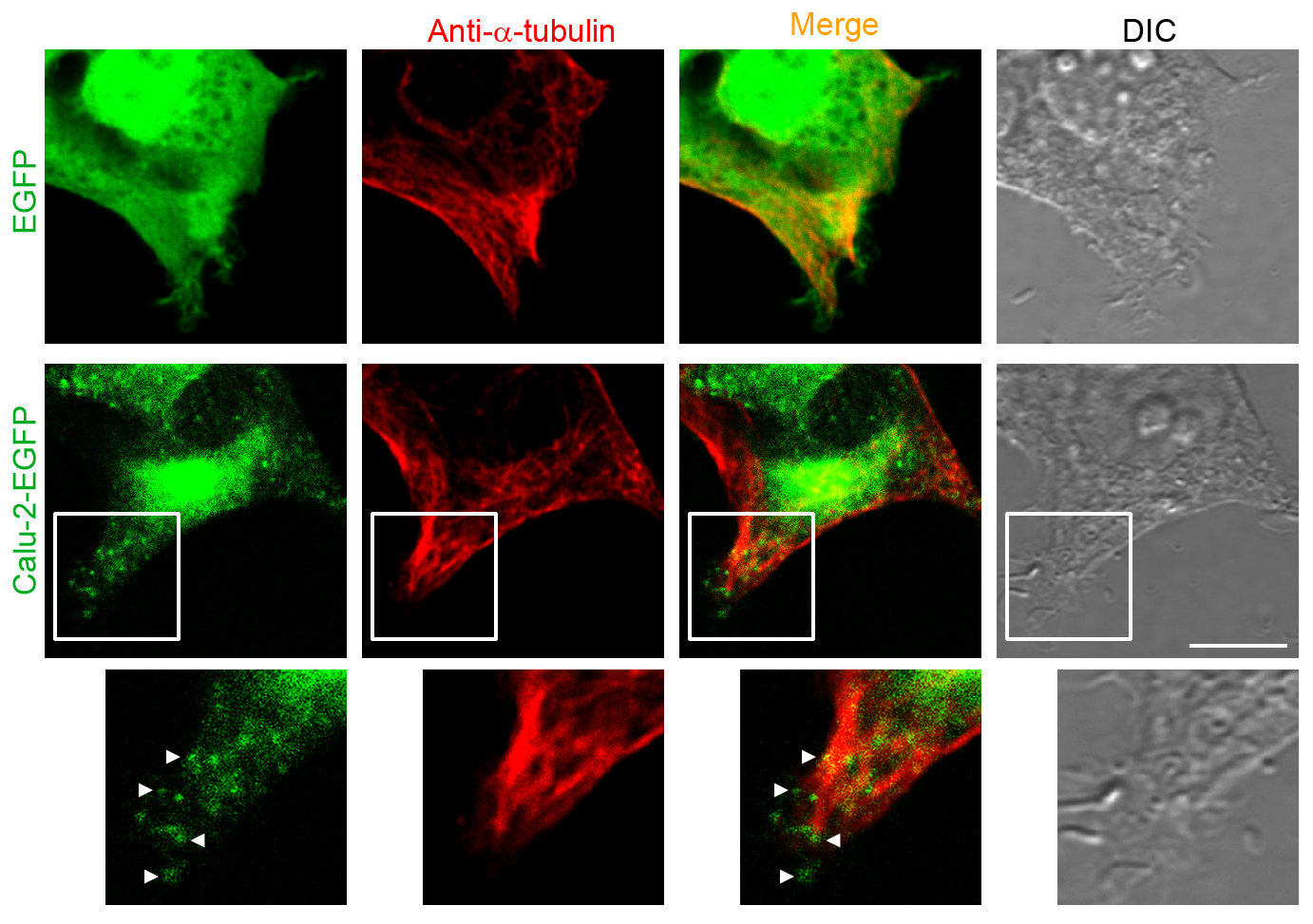

Supplement: Figure S3 — Immunofluorescence of EGFP or Calu-2-EGFP overexpressing HEK293T cells probed with anti-α-tubulin antibody. The rectangle areas were magnified. Scale bar, 10 µm. (TIF) [file pone.0035344.s003.tif]
