# Supplementary material for: The Intracellular Transport and Secretion of Calumenin-1/2 in Living Cells
Source: PLoS One. 2012 Apr 13;7(4):e35344. doi: 10.1371/journal.pone.0035344 (PMC3325945; doi:10.1371/journal.pone.0035344)
Supplement: Table S1 — Sequence information of primers used. (DOC) [file pone.0035344.s004.doc]

| Primers used for vector construction of calu-1/2 transcripts | | |
| --- | --- | --- |
| F-calu-1/2-1-EcoRI | GGG GAATTC ATGGACCTGCGACAGTTTCTTATG | |
| R-calu-2-46-SalI | GGG GTCGAC ATAATCAAAACTCTGAGCATCATTGT | |
| F-calu-2-47-EcoRI | GGG GAATTC ATG GACCATGATGCCTTCTTGGGTG | |
| R-calu-2-136-SalI | GGG GTCGAC GTAAGTGCCATAAGTCACGTTTCT | |
| F-calu-2-137-EcoRI | GGG GAATTC ATG CTGGATGATCCAGATCCTGATGAT | |
| R-calu-2-220-SalI | GGG GTCGAC ATGGCTGTACATGTCACCAATATAC | |
| F-calu-2-221-EcoRI | GGG GAATTC ATG GATGGGAATACTGATGAGCCAGAA | |
| R-calu-1/2-315-SalI | GGG GTCGAC GAACTCATCATGCCGTACTAAGG | |
| F-calu-2-19-EcoRI | AATTC ATGGACCTGCGACAGTTTCTTATGTGCCTGTCCCTGTGCACAGCCTTTGCCTTGAGC G | |
| R-calu-2-19-SalI | TCGAC GCTCAAGGCAAAGGCTGTGCACAGGGACAGGCACATAAGAAACTGTCGCAGGTCCAT G | |
| F-calu-2-22-EcoRI | AATTC ATGGACCTGCGACAGTTTCTTATGTGCCTGTCCCTGTGCACAGCCTTTGCCTTGAGCAAACCCACA G | |
| R-calu-2-22-SalI | TCGAC TGTGGGTTTGCTCAAGGCAAAGGCTGTGCACAGGGACAGGCACATAAGAAACTGTCGCAGGTCCAT G | |
| F-calu-2Δ4 | GTGCACAGCCTTTGCCTTGAGC GATGGGAATACTGATGAGCCAGAA | |
| R-calu-2Δ4 | TTCTGGCTCATCAGTATTCCCATC GCTCAAGGCAAAGGCTGTGCAC | |
| F-calu-2Δ5 | GTGCACAGCCTTTGCCTTGAGC TACCTGGATGATCCAGATCCTGAT | |
| R-calu-2Δ5 | ATCAGGATCTGGATCATCCAGGTA GCTCAAGGCAAAGGCTGTGCAC | |
| F-calu-2Δ6 | GTGCACAGCCTTTGCCTTGAGC GACCATGATGCCTTCTTGGGTGC | |
| R-calu-2Δ6 | GCACCCAAGAAGGCATCATGGTC GCTCAAGGCAAAGGCTGTGCAC | |
|  | | |
| Primers used for vector construction of calu-2 point mutations | | |
| F2-calu-2-P21A | GCCTTGAGCAAAGCCACAGAAAAG | |
| R2-calu-2-P21A | CTTTTCTGTGGCTTTGCTCAAGGC | |
| F2-calu-2-N131A | GAGTACAGAGCCGTGACTTATGGC | |
| R2-calu-2-N131A | GCCATAAGTCACGGCTCTGTACTC | |
|  | | |
| Primers used for vector construction of Kif5b and p50 | | |
| F-Kif5b-KLCBD-EcoRI | | GGG GAATTC AATG GAACGTCTAAGAGTAGAACATGAGA |
| R-Kif5b-KLCBD-SaI | | AAA GTCGAC TTA GCCTCCGGTGTCATCAGAATCAA |
| F-Kif5b-CBD-EcoRI | | GGG GAATTC AATG AGCGCTGCTCAGAAGCAAAAAATC |
| R-Kif5b-CBD-SaI | | AAA GTCGAC TTA CACTTGTTTGCCTCCTCCACCT |
| F-p50-EcoRI | | AAA GAATTC ATGGCGGACCCTAAATACGC |
| R-p50-SalI | | AAA GTCGAC ACTGTGGCCAGGATTTTCAC |
|  | |  |
